# Animal Cell Cytokinesis: The Rho-Dependent Actomyosin-Anilloseptin Contractile Ring as a Membrane Microdomain Gathering, Compressing, and Sorting Machine

**DOI:** 10.3389/fcell.2020.575226

**Published:** 2020-10-07

**Authors:** Sabrya C. Carim, Amel Kechad, Gilles R. X. Hickson

**Affiliations:** ^1^CHU Sainte-Justine Research Center, Université de Montréal, Montréal, QC, Canada; ^2^Département de Pathologie et Biologie Cellulaire, Faculté de Médecine, Université de Montréal, Montréal, QC, Canada

**Keywords:** cytokinesis, anillin, septin, membrane microdomains, membrane cytoskeleton, rho (Rho GTPase), contractile ring tension, contractile ring mechanism

## Abstract

Cytokinesis is the last step of cell division that partitions the cellular organelles and cytoplasm of one cell into two. In animal cells, cytokinesis requires Rho-GTPase-dependent assembly of F-actin and myosin II (actomyosin) to form an equatorial contractile ring (CR) that bisects the cell. Despite 50 years of research, the precise mechanisms of CR assembly, tension generation and closure remain elusive. This hypothesis article considers a holistic view of the CR that, in addition to actomyosin, includes another Rho-dependent cytoskeletal sub-network containing the scaffold protein, Anillin, and septin filaments (collectively termed anillo-septin). We synthesize evidence from our prior work in *Drosophila* S2 cells that actomyosin and anillo-septin form separable networks that are independently anchored to the plasma membrane. This latter realization leads to a simple conceptual model in which CR assembly and closure depend upon the micro-management of the membrane microdomains to which actomyosin and anillo-septin sub-networks are attached. During CR assembly, actomyosin contractility gathers and compresses its underlying membrane microdomain attachment sites. These microdomains resist this compression, which builds tension. During CR closure, membrane microdomains are transferred from the actomyosin sub-network to the anillo-septin sub-network, with which they flow out of the CR as it advances. This relative outflow of membrane microdomains regulates tension, reduces the circumference of the CR and promotes actomyosin disassembly all at the same time. According to this hypothesis, the metazoan CR can be viewed as a membrane microdomain gathering, compressing and sorting machine that intrinsically buffers its own tension through coordination of actomyosin contractility and anillo-septin-membrane relative outflow, all controlled by Rho. Central to this model is the abandonment of the dogmatic view that the plasma membrane is always readily deformable by the underlying cytoskeleton. Rather, the membrane resists compression to build tension. The notion that the CR might generate tension through resistance to compression of its own membrane microdomain attachment sites, can account for numerous otherwise puzzling observations and warrants further investigation using multiple systems and methods.

## Introduction

Cytokinesis of animal cells begins during anaphase with the formation of a cleavage furrow at the cell equator, between the separating chromosomes. At the base of this furrow is a “contractile ring” (CR) (Schroeder, 1972, 1990) that exerts force (Rappaport, 1977) to dramatically reduce the circumference of the cell’s plasma membrane and form a midbody ring, which then orchestrates abscission. Pioneering electron microscopy of sea urchin embryos by Schroeder showed that the CR contains circumferential actin filaments (Schroeder, 1972). This was quickly followed by studies showing that the CR contains myosin (Schroeder, 1973; Fujiwara and Pollard, 1976; Mabuchi and Okuno, 1977). It was initially thought that the CR might have a muscle sarcomere-like organization and close like a purse-string. However, the actin filaments of the CR are not organized as in sarcomeres, but are rather organized as a meshwork of filaments in a random order of mixed polarity (Sanger and Sanger, 1980; Fishkind and Wang, 1993; Mabuchi, 1994; Mabuchi et al., 1988; Kamasaki et al., 2007; Spira et al., 2017). An additional class of cytoskeletal filaments, the septins, was discovered as membrane-associated filaments of the budding yeast CR (Byers and Goetsch, 1976; Haarer and Pringle, 1987). Septins are conserved components of CRs from yeast to humans, yet the roles that they play there and how they interact with actomyosin filaments remain unclear (Caudron and Barral, 2009; Mostowy and Cossart, 2012; Bridges and Gladfelter, 2015; Addi et al., 2018; Marquardt et al., 2019). It has been evident since the work of Schroeder (Schroeder, 1972), and more recently Carvalho (Carvalho et al., 2009), that the CR disassembles as it closes, since its closure results in a decrease in its circumference but does not increase its thickness or width. From many studies since these pioneering works, we have learned much about the CR, its constituent parts and their biochemical activities (Eggert et al., 2006; Green et al., 2012). Yet we still do not understand how those components are organized and coordinated with one another to promote CR assembly and closure, particularly in animal cells. Indeed, some of the most enduring and fundamental questions in the field that remain unanswered include: How does the CR generate tension? How does the CR maintain tension while closing and disassembling at the same time? How is the CR anchored to the plasma membrane? How are the different filament systems of the CR (F-actin, myosin II, septins) coordinated with one another (Green et al., 2012; Pollard, 2017; Gerien and Wu, 2018; O’Shaughnessy and Thiyagarajan, 2018; Leite et al., 2019; Mangione and Gould, 2019; Pollard and O’Shaughnessy, 2019)? This article seeks to address these questions. The following sections will briefly summarize the current state of knowledge of the key players involved in CR assembly and function, before describing novel models for how the actomyosin and septin sub-networks of the CR collaborate to generate tension during CR assembly and regulate it to drive CR closure.

### Rho: The Master Activator of Cytokinesis

The small molecular weight GTPase Rho has emerged as the master activator of cytokinesis, and specifically, CR assembly. From the onset of cytokinesis, RhoA (Rho1 in *Drosophila*) undergoes dynamic cycles of activation/inactivation at the equatorial plasma membrane (Drechsel et al., 1997; Bement et al., 2005, 2015; Miller et al., 2008; Miller and Bement, 2009; Basant and Glotzer, 2018) with activation through GDP to GTP exchange catalyzed by a specific guanine nucleotide exchange factor (GEF), ECT2 (Miki et al., 1993; Tatsumoto et al., 1999; Kimura et al., 2000) Pebble in *Drosophila* (Lehner, 1992; Somers and Saint, 2003) and inactivation promoted by GTP hydrolysis catalyzed by GTPase activating proteins (GAPs). ECT2/Pebble forms a complex with the centralspindlin complex (Somers and Saint, 2003; Yuce et al., 2005; Kamijo et al., 2006; Nishimura and Yonemura, 2006), comprising kinesin-6 (MKLP1/KIF23 in mammals, Pavarotti in *Drosophila*, ZEN4 in *C. elegans*) and RacGAP (MgcRacGAP in mammals, Tumbleweed/RacGAP50C in *Drosophila*, CYK4 in *C. elegans*), which is required both to activate the GEF activity (via the RacGAP component) and localize it to the equator (via the kinesin-6 component) (Glotzer, 2009; Mishima, 2016). Although centralspindlin-ECT2/Pebble complexes localize to the central spindle at the cell interior, ECT2/Pebble is specifically active as a Rho GEF only at the plasma membrane (Kotynkova et al., 2016; Basant and Glotzer, 2018; Chen M. et al., 2020). There, active Rho-GTP in turn binds and activates specific effector proteins (described below), while RhoGAPs stimulate GTP hydrolysis to rapidly inactivate Rho. Although ECT2/Pebble clearly controls the major Rho-dependent events during cytokinesis, the RhoGAPs responsible for inactivation of specific Rho-effector complexes are less clear. The RacGAP component of the centralspindlin complex required for ECT2/Pebble activation has long been a candidate and its involvement in Rho inactivation is supported by the analysis of GAP-defective MgcRacGAP in *Xenopus* embryos (Miller and Bement, 2009). However, RacGAP exhibits preferential GAP activity toward Rac and Cdc42 rather than Rho *in vitro* (Toure et al., 1998; Bastos et al., 2012; Jantsch-Plunger et al., 2000). Genetic experiments in *C. elegans* embryos have led to conflicting models (discussed in Basant and Glotzer, 2017) in which the CYK4 GAP domain either targets Rac (Canman et al., 2008; Zhuravlev et al., 2017), or acts non-canonically to activate Rho (Loria et al., 2012; Zhang and Glotzer, 2015). Another candidate GAP for Rho inactivation at the CR is p190RhoGAP-A/ARHGAP35 (Mikawa et al., 2008; Su et al., 2009; Manukyan et al., 2015). M-phase GAP (MP-GAP:ARHGAP11A in mammals, RGA-3/4 in *C. elegans*) also acts to globally suppress Rho and restrict cortical contractility to the equator (Zanin et al., 2013), although no ortholog exists in *Drosophila*. Finally, an additional level of regulation of the Rho GTPase cycle comes from guanine nucleotide dissociation inhibitors (GDIs), which can directly extract active Rho from the plasma membrane (Golding et al., 2019).

While our understanding of the dynamic control of Rho activity is far from complete, it is clear that active Rho-GTP binds and activates multiple effectors that are essential for cytokinesis. These include Diaphanous-related formins, which polymerize unbranched actin filaments (Castrillon and Wasserman, 1994; Otomo et al., 2005; Watanabe et al., 2008, 2010, 2013b). Rho-GTP also activates Rho-kinase, which in turn activates myosin II (Kosako et al., 1999, 2000; Piekny and Mains, 2002; Matsumura, 2005; Hickson et al., 2006), through both stimulatory phosphorylation of the myosin regulatory light chain (MRLC) and inhibitory phosphorylation of the myosin phosphatase (Kawano et al., 1999; Matsumura, 2005). Together, these Rho-dependent events provide the textbook description for actomyosin-based CR assembly (Morgan, 2006).

**FIGURE 1:**
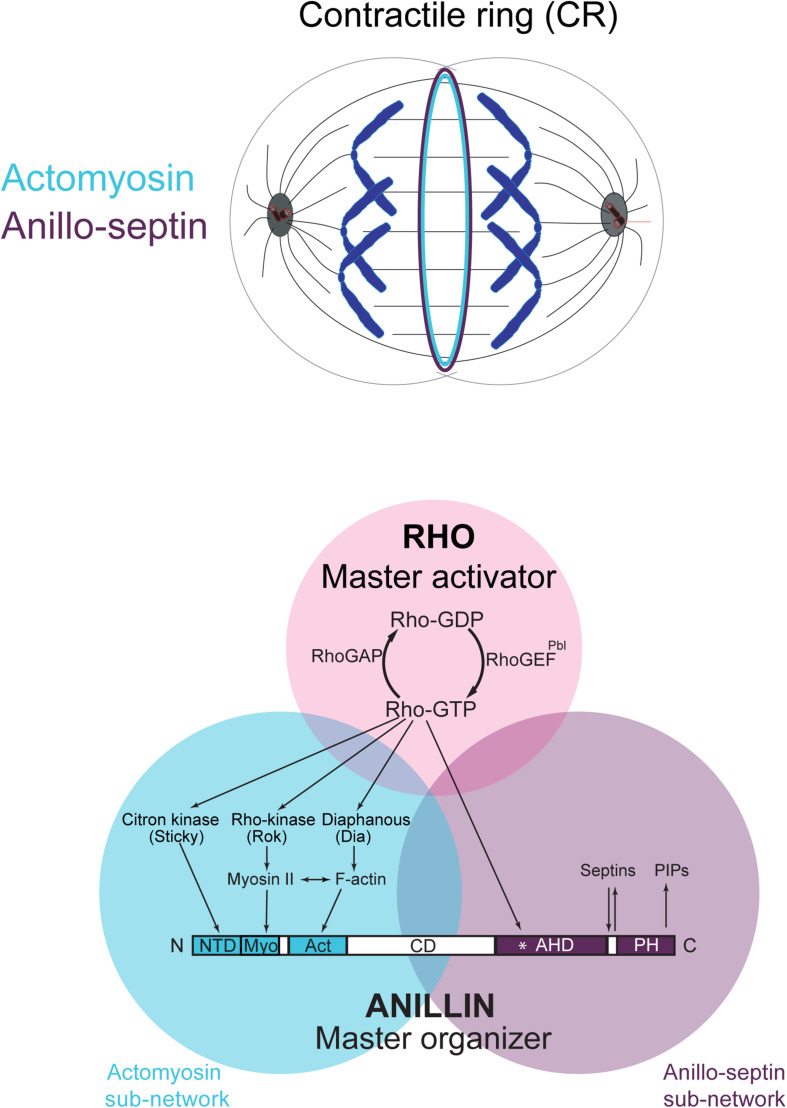
The Rho-dependent network controlling the contractile ring. The Rho GTPase is the master activator of cytokinesis. Rho-GTP activates multiple essential effectors during cytokinesis: Rho kinase, Citron kinase, the formin Diaphanous and the multi-domain scaffold protein, Anillin. Anillin is the master organizer of cytokinesis that can bind many other components of the Rho network and the plasma membrane. Within the contractile ring, Anillin organizes two Rho-dependent cytoskeletal sub-networks: actomyosin and anillo-septin.

However, Rho controls a more expansive network than just formins and Rho-kinase (Figure 1). Rho-GTP also binds and recruits to the CR Anillin (Hickson and O’Farrell, 2008b; Piekny and Glotzer, 2008; Sun et al., 2015; El-Amine et al., 2019) and Citron kinase (called Sticky in *Drosophila* (Madaule et al., 1995, 1998; Di Cunto et al., 1998; Eda et al., 2001; Gai et al., 2011; Watanabe et al., 2013a; El-Amine et al., 2019). As described in the next section, Anillin is a conserved scaffold protein that has emerged as a master organizer of the CR (reviewed in Hickson and O’Farrell, 2008a; D’Avino, 2009; Piekny and Maddox, 2010). Citron kinase/Sticky does not appear to play important roles at the CR but becomes essential for formation of the subsequent midbody ring (MR) (Di Cunto et al., 2002; D’Avino et al., 2004; Echard et al., 2004; Naim et al., 2004; Dean and Spudich, 2006; Bassi et al., 2011, 2013; Gai et al., 2011; El Amine et al., 2013, El-Amine et al., 2019; Watanabe et al., 2013a; McKenzie et al., 2016; D’Avino, 2017; Dema et al., 2018). The MR is a stable cortical ring structure that forms after CR closure at the center of the intercellular bridge, where it encircles the densely packed zone of interdigitating microtubule plus-ends of the midbody (Steigemann and Gerlich, 2009; Green et al., 2012; Hu et al., 2012; D’Avino et al., 2015). Although the mechanisms of MR formation are poorly defined, MRs contain numerous CR-derived proteins including Anillin, Citron kinase/Sticky, myosin, centralspindlin, as well as CEP55 in mammalian cells. The MR persists after the midbody microtubules have depolymerized and it recruits ESCRT proteins to mediate abscission (Carlton and Martin-Serrano, 2007; Elia et al., 2011; Guizetti et al., 2011).

Thus Rho activates at least four downstream effector pathways that must collaborate at the CR and MR: the formin Diaphanous, Rho-kinase, Citron kinase and Anillin.

### Anillin: The Master Organizer of Cytokinesis

Anillin is an integral component of all cytokinetic CRs (Field and Alberts, 1995; Giansanti et al., 1999). Through conserved domains, it can bind a host of other essential CR components, including F-actin, which it bundles (Field and Alberts, 1995; Oegema et al., 2000; Tian et al., 2015; Jananji et al., 2017) and myosin II (Straight et al., 2005). Accordingly, Anillin is often framed as an actomyosin crosslinker. However, Anillin can also bind many other components including Rho-GTP (Sun et al., 2015; Budnar et al., 2019), Citron kinase/Sticky (Gai et al., 2011; El-Amine et al., 2019), membrane phospholipids (Liu et al., 2012) and septins (Oegema et al., 2000; Kinoshita et al., 2002; Figure 1).

Septins form palindromic, hetero-oligomeric rod-like complexes that polymerize end-on to form non-polar filaments that can in turn assemble into higher-ordered structures (Sirajuddin et al., 2007; Mostowy and Cossart, 2012; Bridges and Gladfelter, 2015; Spiliotis, 2018; Mendonça et al., 2019; Soroor et al., 2019). In *Drosophila*, the prototypic complex is a hexamer comprising two copies each of Peanut (mammalian SEPT7 ortholog), Sep1 (mammalian SEPT2) and Sep2 (mammalian SEPT1) (Field et al., 1996). According to the recent data of Mendonça and Soroor, the order of the hexamer is likely Sep1-Sep2-Peanut-Peanut-Sep2-Sep1. Septins bind PIP2 (Zhang et al., 1999; Tanaka-Takiguchi et al., 2009; Mendonça et al., 2019; Soroor et al., 2019) which stimulates septin polymerization (Bertin et al., 2010) and septin filaments are often found in tight association with the plasma membrane, where they can form membrane diffusion barriers (Takizawa et al., 2000; Dobbelaere and Barral, 2004; Caudron and Barral, 2009) and contribute to cortical rigidity (Gilden and Krummel, 2010; Mostowy and Cossart, 2011). In both worm and fly cells, the recruitment of septins to the CR depends on Anillin (Maddox et al., 2005; Hickson and O’Farrell, 2008b). Yet, despite conservation from yeast to humans, the precise roles played by the septin cytoskeleton at the CR remain obscure.

**FIGURE 2:**
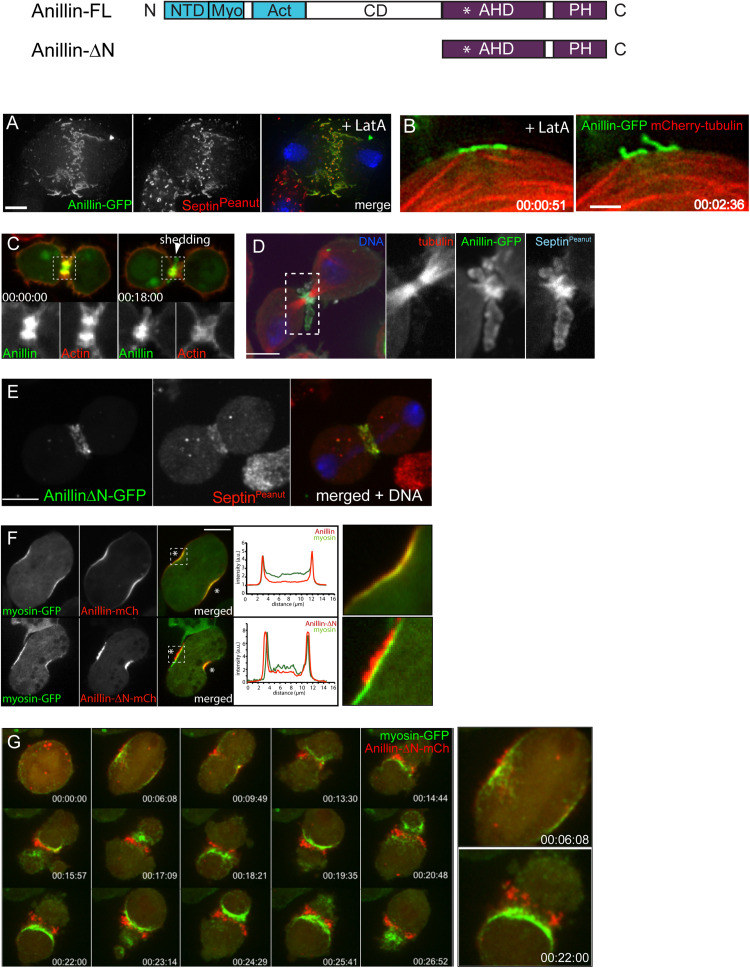
Actomyosin and anillo-septin are independently anchored to the plasma membrane and can laterally separate from one another. **(A)** Rho1 drives assembly of anillo-septin structures directly at the equatorial plasma membrane in the presence of the F-actin inhibitor, Latrunculin A. Maximum intensity projection of a fixed S2 cell expressing Anillin-GFP, stained for endogenous septin (Peanut) and DNA. Similar behavior of anillo-septin is observed upon additional inhibition of myosin activation (not shown). **(B)** Actin-independent assembly of anillo-septin sequesters the plasma membrane. Time-lapse frames of close-ups of the cell cortex of an S2 cell co-expressing Anillin-GFP (green) and mCherry-tubulin (red) treated with Latrunculin A and progressing through anaphase. The nascent anillo-septin structures reorient from parallel to perpendicular to the cell surface as they envelop themselves in plasma membrane. This envelopment and reorientation suggest that, as the anillo-septin complexes bind and sequester the membrane microdomains, the resulting tubular structures can deform the membrane to make it fit their shape. Time is shown as h:min:s from anaphase, scale bar, 2 μm. **(C)** Anillin-mCherry (mCh, green) shedding from the late contractile ring/nascent midbody ring (indicated by the white arrowhead) of a *Drosophila* S2 cell co-expressing the F-actin probe Lifeact-GFP (red), which does not shed). Split channels of the dashed boxed region are shown magnified below. **(D)** Shed material contains Anillin and Peanut. Fixed cells expressing Anillin-GFP (green), mCh-tubulin (red) and stained for endogenous Peanut (cyan) and DNA (Hoechst, blue). **(E)** The Anillin C-terminus (Anillin-ΔN) and the septin, Peanut, are co-recruited to the CR. Fixed *Drosophila* S2 cell expressing Anillin-ΔN-GFP, stained for endogenous Peanut and DNA (Hoechst, blue). **(F)** *Drosophila* S2 cell during early furrowing co-expressing myosin-GFP (green) and either Anillin-mCherry (top) or the Anillin C-terminus (Anillin-ΔN-mCherry, red, bottom). Intensity profiles along a line drawn between the asterisks are shown and white dashed boxed regions are shown magnified on the right. While Anillin-mCherry localization closely follows that of myosin-GFP, Anillin-ΔN-mCherry appears in puncta that extrude outward toward the cell exterior. **(G)** Frames from a time-lapse sequence of an S2 cell co-expressing myosin-GFP (green) and Anillin-ΔN-mCh (red) and depleted of endogenous Anillin. Anillin-ΔN (anillo-septin) forms punctate structures that herniate the membrane outward, whilst myosin (actomyosin) undergoes back-and-forth, lateral oscillations beneath these distinct structures. This clearly shows that actomyosin and anillo-septin are separate from one another (at least when the Anillin N-terminus is missing) and that they are independently anchored to the plasma membrane. The 00:06:08 and 00:22:00 time points are shown magnified on the right. Times are h:min:s from anaphase. **(A)** adapted and **(B)** reproduced from © 2008 Hickson and O’Farrell originally published in Journal of Cell Biology: https://doi.org/10.1083/jcb.200709005 which is available under a Creative Commons License (Attribution–Non-commercial–Share Alike 4.0 Unported license, as described at http://creativecommons.org/licenses/by-nc-sa/4.0/) and for which we retain copyright (Hickson and O’Farrell, 2008b). **(C,D)** adapted from© 2013 El-Amine et al., originally published in Journal of Cell Biology: https://doi.org/10.1083/jcb.201305053, which is available under a Creative Commons License (Attribution–Non-commercial–Share Alike 3.0 Unported license, as described at http://creativecommons.org/licenses/by-nc-sa/3.0/) and for which we retain copyright (El Amine et al., 2013). **(E–G)** are adapted from Kechad et al. (2012), originally published in Current Biology with permission from Elsevier (publisher) license number: 4844230307942 (obtained on June 08th, 2020) https://doi.org/10.1016/j.cub.2011.11.062.

Over the past 12 + years, we have studied the Rho1-dependent cytokinetic network in *Drosophila* S2 cells, with an emphasis on Anillin function. Our work has revealed that Anillin (Kechad et al., 2012; El Amine et al., 2013), together with its binding partner Citron kinase/Sticky mediates the “CR-to-MR transition” (El Amine et al., 2013, El-Amine et al., 2019). Rather than the traditional view of the MR and CR as entirely separate structures, our work suggests that Anillin promotes the maturation of the CR into the MR. Indeed, analysis of truncation mutants revealed that Anillin can localize to and organize two separable, Rho1-dependent sub-networks: the actomyosin cytoskeleton via its N-terminus, and the membrane-anchored septin cytoskeleton via its C-terminus (Kechad et al., 2012; El Amine et al., 2013). We further showed that the CR-to-MR transition reflects a balance between two opposing mechanisms acting simultaneously on Anillin: one of cortical retention (mediated by the Anillin N-terminus and Citron kinase/Sticky), the other of membrane-associated removal (mediated by the Anillin C-terminus and septins, Kechad et al., 2012; El Amine et al., 2013) and Figures 2C,D. These works not only demonstrate the central importance of Anillin during cytokinetic progression, but they also highlight how Anillin can be used as a tool to break down the complex cytokinetic network into more manageable and observable sub-networks.

In this *Hypothesis and Theory* article, we revisit several key observations that, together with multiple iterations of “night science” (Yanai and Lercher, 2019), have led us to a more global view of the CR than is often described. A more expansive view considers not only actomyosin, but also Anillin, septins and, crucially, the plasma membrane microdomain attachment sites to which all of these cytoskeletal elements are anchored. We do not attempt to provide an exhaustive review of the literature on the CR, as this has already been expertly done recently (Glotzer, 2017; O’Shaughnessy and Thiyagarajan, 2018; Leite et al., 2019; Mangione and Gould, 2019; Pollard and O’Shaughnessy, 2019). We rather seek to expose a novel conceptual framework, in which the plasma membrane plays a much more active role in regulating the tension of the CR than has generally been considered. The insight gained from our Anillin-centric experimental approach leads to a simple yet compelling model for how the global Rho GTPase network and the plasma membrane influence one another during CR assembly and closure.

### Organization of the CR Involves Two Rho-Dependent Sub-Networks

Determining the organization of CRs by direct visualization is challenging because of dynamicity, the degree of compaction of components, and the limits of resolution afforded by light microscopy. In-built redundancy and robustness of the components have also hampered the dissection of the molecular circuitry of the system. However, we found that Anillin truncations can be used as tools to effectively split the CR into two separable and resolvable sub-structures, representing two distinct Rho-dependent sub-networks that Anillin ordinarily localizes to: an actomyosin-based sub-network and a septin-based sub-network (Figure 1; Kechad et al., 2012; El Amine et al., 2013). Considering the CR as a dynamic marriage of these two Rho-dependent, Anillin-organized sub-networks is what led to the model elaborated below.

### A Rho-Dependent Actomyosin Sub-Network Organized by the Anillin N-Terminus

During CR formation Rho activation drives actomyosin assembly through formin-mediated F-actin polymerization and Rho-kinase-dependent myosin II activation. Actomyosin-driven CRs can assemble without Anillin, but such Anillin-deficient CRs are defective. In fly and human cells, Anillin-depleted CRs fail to close completely, and become unstable and prone to lateral oscillation (Straight et al., 2005; Zhao and Fang, 2005; Hickson and O’Farrell, 2008b; Piekny and Glotzer, 2008; Goldbach et al., 2010; Kechad et al., 2012). In larger *C. elegans* zygotes, Anillin-depleted CRs successfully close but they are abnormally sensitive to reduced myosin levels (Maddox et al., 2007) and they exhibit higher levels of F-actin (Jordan et al., 2016). But although the actomyosin elements of the CR can assemble independently of Anillin, Anillin does connect to this actomyosin sub-network and it does so using its N-terminal half. This region harbors conserved domains for binding F-actin (Field and Alberts, 1995; Tian et al., 2015; Jananji et al., 2017; Matsuda et al., 2020) and myosin II (Straight et al., 2005), and the Anillin N-terminus (Anillin-ΔC) is sufficient to co-localize with actomyosin at the CR in a similar manner to full-length Anillin (e.g., Kechad et al., 2012; El Amine et al., 2013). Disrupting the actin cytoskeleton with Latrunculin A (LatA) disrupts the cortical localization of the Anillin N-terminus (Anillin-ΔC) during anaphase (unpublished data), indicating that F-actin is required to recruit the Anillin N-terminus to the actomyosin sub-network of the CR. Within that sub-network, Anillin’s F-actin cross-linking ability may contribute to tension generation within the CR (Field and Alberts, 1995; Tian et al., 2015; Jananji et al., 2017; Matsuda et al., 2020) and it also enhances formin-mediated F-actin polymerization (Chen et al., 2017). Anillin−myosin interactions presumably also play a role at the CR (Straight et al., 2005), although these have not been directly tested. Finally, interactions with Dia-related formin (Watanabe et al., 2010) and Citron-kinase/Sticky (El Amine et al., 2013, El-Amine et al., 2019) that also reportedly occur in the Anillin N-terminus, likely also impinge on this actomyosin-based sub-network, which the Anillin N-terminus coordinates. Regardless of any other potential contributions, the N-terminal half of Anillin, and by extension the actomyosin sub-network that it participates in, can only provide partial functionality to the CR. Indeed, although expressing the Anillin N-terminus (Anillin-ΔC) in *Drosophila* S2 cells depleted of endogenous Anillin rescued the oscillating CR phenotype (the only animal system where, to our knowledge, such a construct has been examined), the resulting Anillin N-terminus-organized CRs closed slowly and displayed aberrant membrane blebbing in their flanking furrows (Kechad et al., 2012). Thus, for their proper function, CRs must require additional Anillin-dependent activities associated with its C-terminus.

### A Rho-Dependent Anillo-Septin Sub-Network Organized by the Anillin C-Terminus

Anillin recruitment is also Rho-dependent, and the requirement for ECT2/Pebble indicates that “active Rho” is required (Hickson and O’Farrell, 2008b; Piekny and Glotzer, 2008). This recruitment mechanism occurs via the C-terminus of Anillin (Piekny and Glotzer, 2008; Kechad et al., 2012), which contains a conserved Rho binding domain within its Anillin Homology Domain (Piekny and Glotzer, 2008; Tse et al., 2012; Sun et al., 2015; Nishikawa et al., 2017; Michaux et al., 2018; Budnar et al., 2019), as well as a PH domain that can bind both membrane phospholipids (Liu et al., 2012) and septins (Oegema et al., 2000; Field C.M. et al., 2005). Indeed, Anillin is required to recruit septins to the CR in *C. elegans* embryos (Maddox et al., 2005), fly cells and embryos (Field C.M. et al., 2005; Hickson and O’Farrell, 2008b), and human cells via a region that includes the PH domain (Liu et al., 2012). Reciprocally, robust recruitment of the fly C-terminal AH/PH domains (Anillin-ΔN) depends on septins (Kechad et al., 2012), indicating that this region of Anillin is more than a simple Rho-GTP binding domain.

The existence of a distinct active-Rho, Anillin and septin sub-network is particularly evident in cells in which the actin cytoskeleton has been completely disrupted using LatA (Hickson and O’Farrell, 2008b). In this case, Rho1, Anillin and septins are co-recruited to the plasma membrane, independently of actomyosin, where they form long tubular structures that become enveloped in plasma membrane, consistent with avid membrane binding (Figures 2A,B; Hickson and O’Farrell, 2008b). For simplicity, and in keeping with the accepted convention of the term “acto-myosin,” we will subsequently use the term “anillo-septin” to describe the Anillin and septin sub-network of the CR.

Thus, in addition to active Rho promoting actomyosin assembly (which can occur independently of anillo-septin), active Rho also promotes anillo-septin assembly (which can occur independently of actomyosin). These separable sub-networks must somehow coexist, interwoven within the CR.

### Actomyosin and Anillo-Septin Are Independently Anchored to the CR Membrane and Can Separate From One Another

**FIGURE 3:**
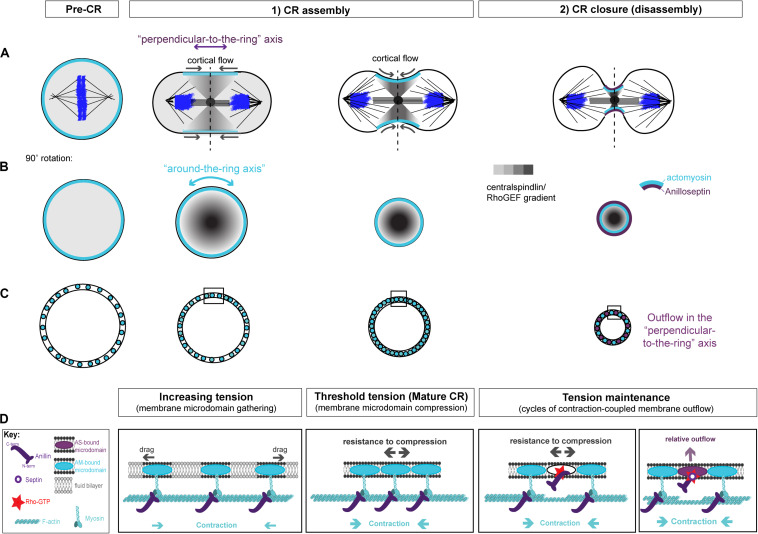
Two-stage model for CR assembly and closure. **(A)** Cartoons of the conventional view of cytokinesis in the “perpendicular-to-the-ring” axis, showing how the architecture of the spindle establishes a gradient of centralspindlin/RhoGEF that peaks at the equator and at the cell center to promote cortical flow during CR assembly and closure. **(B)** 90° rotations of the dotted lines in panel **(A)** showing the “around-the-ring” axis. **(C)** Cartoons in the “around-the-ring” axis to illustrate the proposed concept of how membrane microdomain attachment sites of the CR oppose contractility to generate tension during CR assembly, and regulate tension during CR closure/disassembly. At a certain density of microdomain compaction, which represents a certain tension threshold, the CR becomes “mature” and shifts from the assembly stage to the disassembly stage, which involves relative outflow of microdomains in the “perpendicular-to-the-ring” axis. **(D)** More detailed views of the boxed regions in panel **(C)**, showing the hypothetical arrangement of actomyosin, anillo-septin and their associated membrane microdomains. *Note: The* “*actomyosin sub-network*” *comprises numerous components and membrane anchors* (*not least for F-actin nucleating formins and for myosin II motors*), *but it is purposefully depicted as one entity here for clarity. Similarly, the stoichiometry and scaling of depicted components are for illustrative purposes only.*

Thus, Anillin can localize to both actomyosin (with which it interacts via its N-terminus) and septin-based (via its C-terminus) sub-networks. But crucially, our work shows that Anillin allows these two sub-networks to separate, laterally in the plane of the membrane during CR closure. This physical separation of anillo-septin from actomyosin can be readily observed during the natural progression of the CR-to-MR transition. Curiously, we found that the late CR extrudes and sheds membranes enriched for Rho1-Anillin-septin but lacking actomyosin (El Amine et al., 2013; Figures 2C,D), and others have documented similar phenomena of shedding in other cell types (Mullins and Biesele, 1977; Dubreuil et al., 2007; Renshaw et al., 2014). However, an analogous separation of anillo-septin from actomyosin can also be observed earlier during CR assembly and ingression, upon the experimental truncation of Anillin. While full-length Anillin (or the Anillin N-terminus, Anillin-ΔC) faithfully tracked with the actomyosin components of the CR during the early stages of furrow ingression (Figure 2F), the truncated Anillin C-terminus (Anillin-ΔN) was recruited to the equatorial plasma membrane, independently of actomyosin. Indeed, it was recruited together with Rho1- and septins (Figure 2E) to punctate, membrane-associated structures that rapidly resolved themselves during furrow ingression as being distinct from the actomyosin network, and from which they herniated outward (Figures 2F,G; Kechad et al., 2012). This separation was all the more clear in cells depleted of endogenous Anillin: actomyosin formed unstable CRs that oscillated laterally back and forth across the equator, while the punctate anillo-septin structures remained firmly anchored to the equatorial membrane (Figure 2G). Thus membrane-anchored actomyosin and anillo-septin can clearly separate from one another, both naturally (during shedding from the late CR/nascent MR) and experimentally (at the early CR following truncation of the Anillin N-terminus). It is therefore logical to surmise that, in the context of a normal CR, the actomyosin and anillo-septin sub-networks might be independently anchored to neighboring patches of plasma membrane, as depicted in Figure 3D. Because in the resolved structures we never saw actomyosin co-localizing with anillo-septin, this suggests that either the two sub-networks utilize different membrane attachment sites (i.e., membrane microdomains) or that they somehow compete for the same membrane microdomains binding them in a mutually exclusive manner. We strongly favor the latter, for reasons discussed below, but the important point here is that the lateral separation of actomyosin and anillo-septin sub-networks drives the lateral separation of their associated membrane microdomains: i.e., neighboring membrane microdomains within the CR membrane can potentially slide past one another, depending on which cytoskeletal sub-network they are anchored to. Consideration of these findings, leads us to propose a simple two-stage model for cytokinetic furrowing (Figure 3): (1) CR assembly, during which tension is generated to a threshold, and (2) CR closure by disassembly, during which tension is maintained at that threshold.

### A Model for CR Assembly and Tension Generation: Rho-Dependent Actomyosin Contractility Gathers and Compresses Membrane Microdomains at the Cell Equator

We envision that, prior to the initiation of cytokinesis (i.e., at metaphase), the plasma membrane contains specific raft-like microdomains that are evenly dispersed within the more fluid phospholipid bilayer, spaced apart through dynamic connections to the isotropic mitotic actomyosin cortex. During anaphase, Rho activation at the equator is triggered by centralspindlin-delivered ECT2/Pebble, but only at specific membrane microdomains (likely PIP2-rich) that can accept the ECT2 PH domain and promote the activation of its GEF activity (Kotynkova et al., 2016; Chen M. et al., 2020). ECT2-stimulated Rho-GTP then recruits effectors for actin and myosin to individual membrane microdomains. Because of the architecture of the spindle, centralspindlin concentrates at the cell equator, first at the central spindle, but then in a disc that emanates outward toward the cortex. This delivers ECT2/Pebble and therefore Rho-GTP to the plasma membrane, in a gradient that peaks at the equator and trails off toward the poles (Figure 3A). In response to this gradient of Rho activation, the actomyosin sub-network contracts in a graded fashion, maximally at the equator, which initiates cortical flow toward the equator. This cortical flow pulls the actomyosin-attached membrane microdomains into the equator also, where they begin to become compressed, again in a gradient that is maximal at the equator (Figures 3A–C). The cortical flow also reinforces and maintains the equatorial peak of the ECT2/Pebble gradient (and downstream components) that was first established by the spindle. During this initial phase of CR assembly, actomyosin and Anillin are connected to one another via Anillin’s N-terminus. The gradient of actomyosin contractility powers the coalescence of associated membrane microdomain attachment sites, gathering them into an increasingly compact ring at the equator (Figures 3A–C). This gathering of cytoskeletal elements appears conceptually similar to the “search, capture, pull, release” model that accounts for the coalescence of pre-CR nodes in *S. pombe* cytokinesis, and where the Anillin-like Mid1 plays a pivotal role (Vavylonis et al., 2008; Ojkic et al., 2011; Rincon and Paoletti, 2012; Bidone et al., 2014; Mangione and Gould, 2019). It is also similar to the compression feedback described by Khaliullin et al. (2018). However, these models do not satisfactorily account for how the nascent CR might generate tension.

Yet as the CR forms, it must generate tension. Tension can be defined as a strained state resulting from forces acting in opposition to each other. As the actomyosin network contracts and pulls on itself, through the coordination of formin-polymerized F-actin and myosin motors/mini-filaments, it pulls its membrane microdomain attachment sites toward each other, gathering them at the expense of the unbound intervening constituents of the phospholipid bilayer that previously separated them (this intervening membrane may also be extruded as microvillus-like projections). Initially, drag forces oppose this gathering of membrane microdomains (Figure 3D), but as the components become increasingly compacted, we postulate that a much greater force will oppose the actomyosin contractility to generate tension within the CR: that of increasing resistance to compression of the membrane microdomain attachment sites themselves (Figure 3D). According to this idea, tension mounts within the nascent CR membrane and associated cortex in response to intra-membrane resistance to compression, which opposes the actomyosin contractility. An analogy for this proposed mechanism of tension generation can be found in the architecture of a traditional wine barrel, which is made of separate wooden staves held together by metal hoops, all under tension. However, the metal hoops are only under tension because of the resistance to compression of the wooden staves, while the wooden staves are only under tension because of the resistance to expansion of the metal hoops. At the CR, actomyosin acts like a tightening metal hoop (except it is on the inside rather than the outside), while the membrane microdomains to which it is anchored are like the wooden staves, resisting compression. It is the opposition of these forces that generate the tension (Figures 3C,D).

We propose that as the nascent CR gathers a certain density of CR-anchored membrane microdomains, their compression reaches a certain threshold in tension, and can now be considered “mature” (Figures 3C,D). This mature CR now transitions from an assembly stage to a disassembly stage, which drives its full closure (Figures 3C,D). According to this model, the assembly stage of the nascent CR is driven primarily by actomyosin, while Anillin follows, bound to actomyosin via its N-terminus.

### A Model for CR Closure by Disassembly: Actomyosin Contractility Pumps Out Anillo-Septin-Bound Membrane Microdomains to Reduce the Circumference of the CR

At this point it is useful to consider the mature CR from its two axes, as described by Khaliullin et al. (2018): the circumferential “around-the-ring” axis and the “perpendicular-to-the-ring” axis (Figure 3). The cortical and membrane flow described above during CR assembly is best envisioned in the “perpendicular-to-the-ring” axis, while the resistance to compression is best envisioned in the “around-the-ring” axis. While the “CR assembly” stage can account for the initiation of furrowing, full closure of the mature CR requires a reduction in its circumference in the “around-the-ring” axis. To achieve this, we propose that ratchet-like cycles of actomyosin contraction (building tension) are coupled to anillo-septin-bound membrane outflow (releasing tension) as the CR advances toward the cell interior and shrinks in circumference. Together, these opposing activities regulate tension such that it can be maintained at the appropriate threshold as the CR closes. Central to this model is the inference that actomyosin and anillo-septin must share the same pool of membrane microdomains, but in a mutually exclusive manner. Only this can satisfactorily accommodate the facts that CRs operate over a wide range cell sizes, using an equivalent starting cortex, and that the relative proportions of each sub-network remain largely constant throughout closure, regardless of initial cell size (Carvalho et al., 2009; Khaliullin et al., 2018).

Indeed we make the specific prediction that, within the mature CR, actomyosin loses its membrane microdomains to anillo-septin, which sequesters them and allows them to flow out of the CR, depriving the actomyosin of the opportunity to reassemble. The net result of such a cycle is net depolymerization of actomyosin, a progressive reduction in the total number of membrane microdomains within the CR, and a concomitant reduction in the circumference of the CR, i.e., CR closure. A useful analogy of this proposed mechanism of ring closure is the party game of musical chairs, where participants (actomyosin) jog around a circle of chairs (membrane micro-domains) to music. When the music is abruptly stopped, the participants must compete for chairs (of which there is 1 fewer than the number of participants) and the person left standing, is eliminated. The music restarts, the participants jog again and the organizer (anillo-septin) removes a chair. Repeated cycles of elimination ensue until there is only one chair and the winner. According to this analogy, anillo-septin (the organizer) removes membrane microdomains (chairs) away from dynamic actomyosin (participants), resulting in a progressive loss of membrane microdomains and a concomitant reduction in the circumference of the contractile ring (the number of participants and chairs progressively declines and the circle closes in).

**FIGURE 4:**
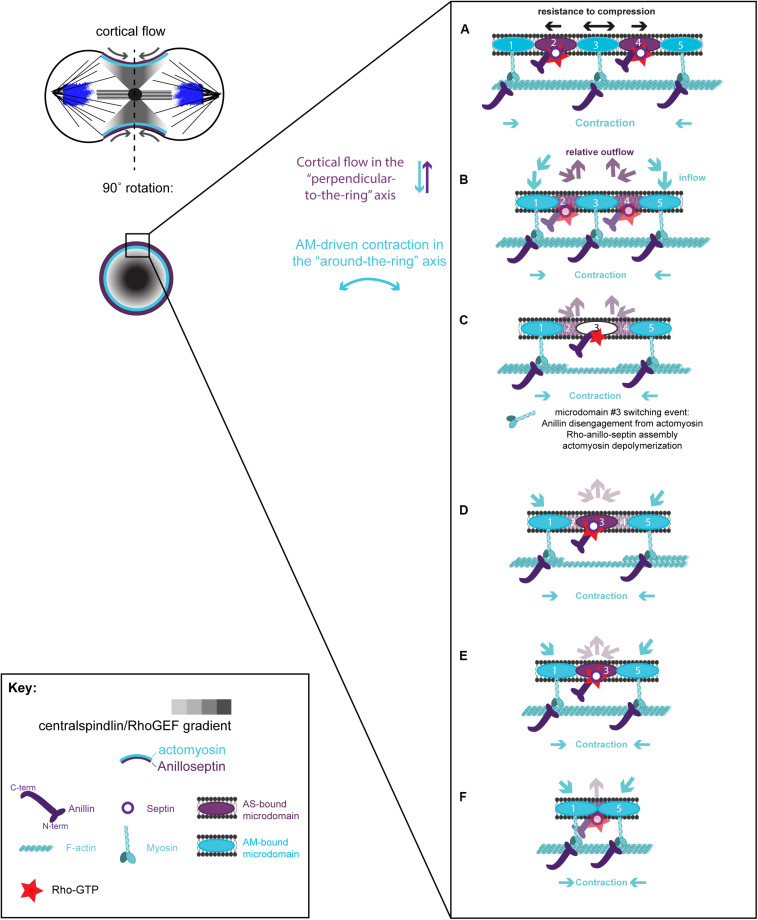
Hypothetical steps in the contraction-coupled anillo-septin membrane sorting model for CR closure, viewed down the spindle/“perpendicular-to-the-ring” axis. **(A)** Actomyosin elements anchored to microdomains 1 and 5 contract in the circumferential, “around-the-ring” axis, compressing the intervening membrane microdomains. Anillin is connected to the membrane either indirectly via actomyosin (microdomains 1, 3, and 5), or directly as anillo-septin (microdomains 2 and 4), but not via both mechanisms at the same time. **(B,C)** Continued contraction advances microdomains 1 and 5 toward the viewer, while the intervening microdomains stay behind (relative outflow). Microdomains 2 and 4 are the latest additions to a growing anillo-septin filament that projects back away from the viewer. Continued contraction pulls microdomains 1 and 5 closer together squeezing them forward, past the intervening microdomains. **(C,D)** Contraction of actomyosin anchored to microdomains 1 and 5, also compresses the intervening actomyosin (reducing its tension) that is anchored to microdomain 3. This in turn allows Rho/Anillin to displace actomyosin (which depolymerizes) and assemble Anillo-septin on microdomain 3, thereby extending the growing anillo-septin filament. **(E,F)** Further contraction pulls microdomains 1 and 5 both closer together and closer to the viewer, past the end of the nascent anillo-septin filament, thereby equilibrating the tension and reducing the circumference of the CR. Thus, the CR closes by contraction-coupled membrane sorting, in which membrane enters and advances the ring with actomyosin but stays behind and exits with anillo-septin. Thus, it disassembles in a bifurcated fashion: actomyosin depolymerization and anillo-septin-membrane outflow.

**FIGURE 5:**
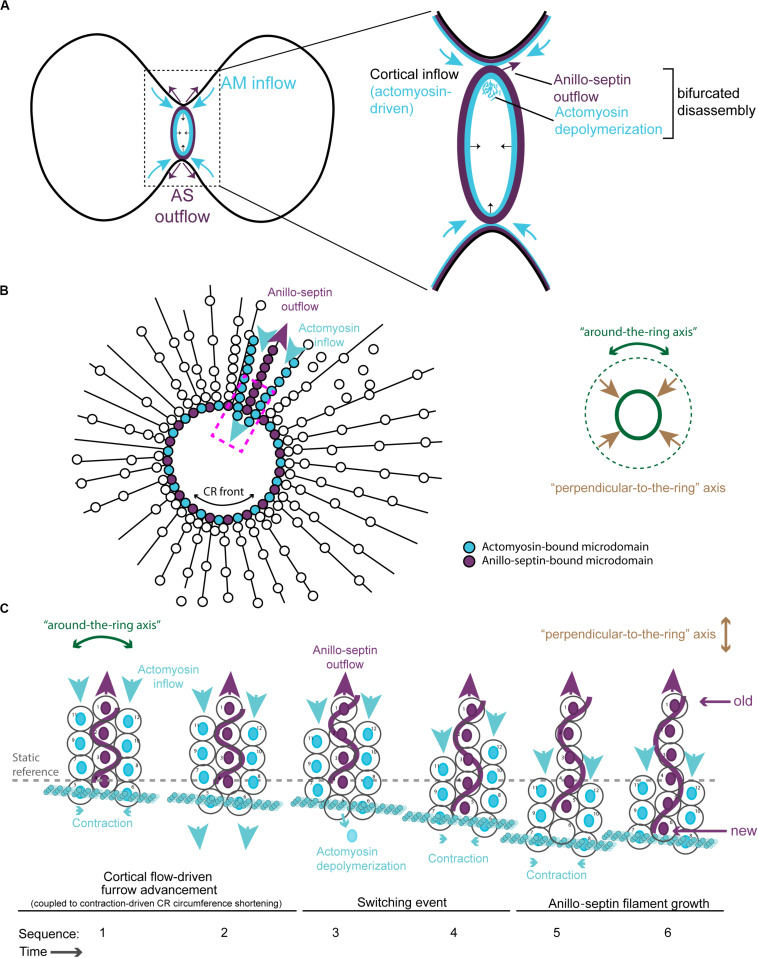
Different viewing perspectives of the contraction-coupled membrane sorting model for CR closure. **(A)** Cartoon showing the proposed relative inflow/outflow of actomyosin/anillo-septin and the concept of closure by bifurcated disassembly, in which the CR sheds components both by depolymerization (actomyosin) and by membrane-anchored outflow (anillo-septin). **(B)** Flattened image viewed down the spindle axis showing both the “around-the-ring” and the “perpendicular-to-the-ring” axes. Arrows depict the relative flow of actomyosin-bound microdomains into the ring (blue) and anillo-septin-bound microdomains out of the ring (purple) as it advances in the “perpendicular-to-the-ring” axis and shrinks in the “around-the-ring” axis. **(C)** Close-up view of the magenta boxed region in panel **(B)**, showing the proposed inflow of actomyosin-bound membrane microdomains, and switching events, as they transfer to nascent anillo-septin filaments that emanate back into the flanks of the furrow. The membrane microdomains are numbered to be able to follow their shifting positions in the time sequence.

A hypothetical series of steps for how the loss of membrane microdomains could operate at the CR is presented in Figure 4. In the first step, contraction in the “around-the-ring” axis of actomyosin-elements bound to two separate membrane microdomains compresses the intervening membrane microdomains. We propose that this compression creates conditions for anillo-septin assembly at the intervening membrane microdomains (Figure 4A) and then squeezes anillo-septin-bound membrane out in the “perpendicular-to-the-ring” axis (Figure 4B). How might this work molecularly? In addition to compressing the intervening membrane microdomains, contraction of actomyosin elements anchored to two membrane microdomains will compress any intervening actomyosin elements that are not directly participating in this contraction event (Figure 4C). Somewhat counter-intuitively for a CR under high tension, this contraction will locally reduce tension on the intervening actomyosin, to which Anillin is bound via its N-terminus. We predict this will provide the opportunity for anillo-septin to assemble on microdomains transiently vacated by actomyosin (Figures 4C,D). Such anillo-septin assembly could potentially involve some sequence of disengagement of Anillin from actomyosin, disengagement of actomyosin from the membrane microdomain, Rho-GTP-mediated recruitment of the Anillin C-terminus and septins to the microdomain, and actomyosin depolymerization (Figure 4C). Whatever the precise mechanism and order of events, anillo-septin assembles on microdomains vacated by actomyosin, thereby sequestering them and providing them the opportunity to exit the ring in the “perpendicular-to-the-ring” axis, as part of a growing, membrane-anchored Anillo-septin filament that is then squeezed out of the ring upon continued contraction of the actomyosin (Figures 4D–F). Anillin is perfectly placed to effectively displace actomyosin from its membrane microdomains because it is bound to actomyosin via its N-terminus, ready to disengage and, via its C-terminus, co-assemble with Rho-GTP and septins at the membrane when the conditions are favorable (i.e., tension-regulated). The predicted relative outflow of anillo-septin-bound membrane microdomains provides a means for regulating the tension within the CR (like a pressure release valve), while the predicted sequestration of shared microdomains prevents re-assembly of actomyosin, therefore promoting net actomyosin disassembly. Thus, CR closure proceeds by a kind of “bifurcated disassembly”: actomyosin depolymerization coupled to anillo-septin-membrane outflow, all operating at a certain threshold in tension (Figure 5A).

According to this model, Anillin flows into the CR bound to actomyosin (but not Rho/septins/membrane), but then exits the CR bound to Rho/septins/membrane (but not actomyosin). From the perspective of the membrane microdomains, they flow into the ring bound to the actomyosin sub-network, then switch to the anillo-septin network (Figures 4C,D), with which they flow out, or rather stay behind in the wake of the CR as it continues its onward journey toward the cell interior. Implicit to the model is the prediction that Anillin’s interactions with Rho/septins (via the C-terminus) and Anillin’s interactions with actomyosin (via the N-terminus) are mutually exclusive, such that Anillin disengages from actomyosin as it switches to the septin network (and displaces actomyosin from its membrane microdomains). For the proposed mechanisms of tension dissipation to be effective, anillo-septin must remain disconnected from actomyosin, at least transiently, to allow the associated membrane microdomains of each sub-network to flow past one another, as this is required to reduce the resistance to compression of the membrane and therefore tension. Super-resolution (fPALM) microscopy of the fission yeast CR cortex has shown it to comprise at least 3 distinct layers with septins occupying the most membrane-proximal layer and F-actin the most membrane-distal layer (McDonald et al., 2017). Perhaps the distance between these layers is ordinarily too great for Anillin to span, until for example an F-actin filament to which it is bound buckles under compression (or depolymerizes), as one expects once the local cortex has reached its threshold in tension. Although highly speculative, this illustrates one of many possibilities for how the system could be organized in a mechanosensitive fashion, regulated by the local tension.

Additional views of the proposed model for CR closure are provided in Figures 5B,C, where the “perpendicular-to-the-ring” axis is projected into a flattened image so that both axes can be visualized simultaneously. These show more clearly the proposed mechanism of actomyosin-dependent inflow of membrane microdomains (blue arrows), and contraction-coupled anillo-septin-dependent outflow of membrane microdomains (purple arrows). The membrane microdomains are color-coded based on the network to which they are attached, to show how they “switch” from the actomyosin sub-network (blue) to the anillo-septin sub-network (purple) at the CR front where the tension is highest (Figures 5B,C). The microdomains are individually numbered to depict their relative movements over a hypothetical time-course in Figure 5C. This arrangement invokes the analogy of mini-caterpillar^TM^ tracks (or conveyor belts) of membrane microdomains flowing forward bound to actomyosin and backward (relatively, i.e., static) bound to anillo-septin. Figure 5C also shows how nascent anillo-septin filaments are predicted to form in the “perpendicular-to-the-ring” axis, with new filament assembly occurring just at/behind the CR front (i.e., the site of maximal tension), utilizing the newly switched membrane microdomains. Thus, while septin filaments are non-polar in the traditional, spatial sense, they may exhibit temporal polarity in cells with “new” ends at the CR and “old” ends projecting toward the poles. To facilitate closure of the CR and prevent it from becoming stuck on itself as the ring circumference shortens, some of these anillo-septin filaments and associated membrane microdomains must presumably disengage from the CR (not shown) and stay behind as the CR advances (i.e., relative outflow).

### The Anillo-Septin Network: Grease for the Actomyosin Network Within the CR?

**FIGURE 6:**
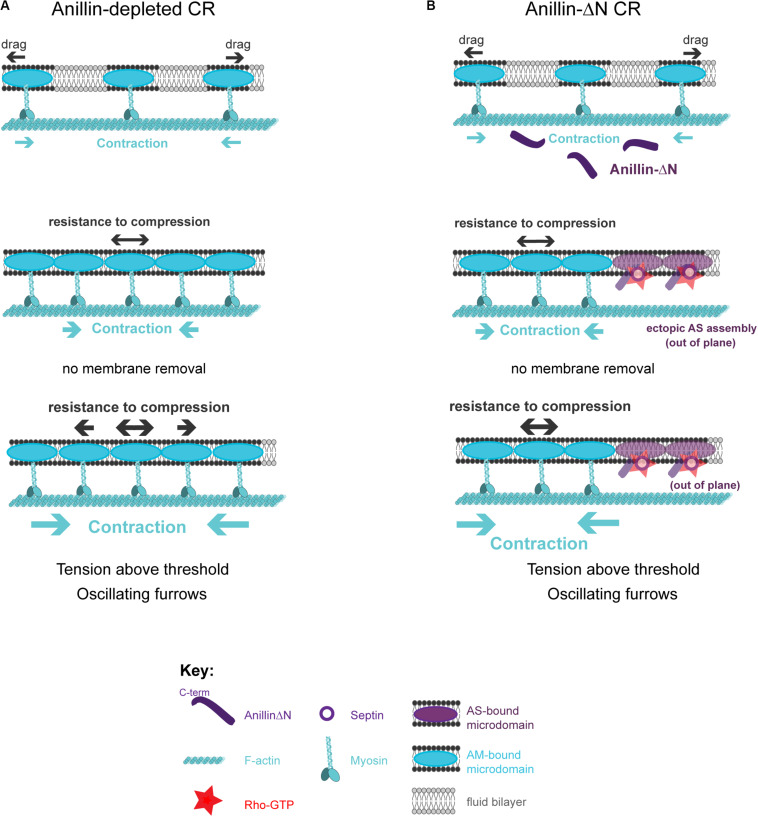
How the proposed model can account for phenotypes observed upon loss of anillo-septin or its coupling to actomyosin. **(A)** Anillin-depleted CR. Actomyosin assembly and contractility persists and is able to gather and compress its own membrane microdomains. However, in the absence of anillo-septin to facilitate membrane microdomain outflow, CR closure stalls because the membrane microdomains impede it and actomyosin cannot disassemble. Tension mounts within the CR to above the usual threshold and this leads to furrow instability and oscillations in the “perpendicular-to-the-ring” axis. **(B)** Deletion of the Anillin N-terminus uncouples anillo-septin from actomyosin. Anillo-septin sequesters membrane, but in a manner that is no longer coupled to actomyosin contractility and at patches of cortex that already trail the CR. Disconnected from Anillin, actomyosin contracts without the means to regulate tension or its own disassembly because it cannot appropriately hand over membrane microdomains to anillo-septin for them to be removed, again leading to stalled CR closure, furrow instability and lateral oscillations.

The described model posits that crosstalk between actomyosin-anilloseptin sub-networks regulates tension and facilitates CR closure through membrane microdomain micro-management. Further support for the model comes from the realization that it provides plausible explanations for otherwise puzzling phenotypes observed upon disruption of the anillo-septin sub-network. Anillin depletion can lead to unstable, oscillating furrows and a failure of CR closure in small cells such as human and fly tissue culture cells (Straight et al., 2005; Zhao and Fang, 2005; Hickson and O’Farrell, 2008b; Piekny and Glotzer, 2008; Kechad et al., 2012) and fly spermatocytes (Goldbach et al., 2010). According to the model, these phenotypes could reflect perturbations in the ability of the CR to sequester and remove its membrane microdomain attachment sites, release tension, disassemble actomyosin and thus close (Figure 6A). Anillo-septin depletion is predicted to lead to unregulated, above-threshold, tension because actomyosin membrane micro-domains accumulate, fail to be extruded, and thus impede CR closure. Continued cortical flow toward this stalling CR could destabilize the CR, potentially by widening it, and lead to its oscillation (Figure 6A). In cells expressing the C-terminus (Anillin-ΔN) as their only source of Anillin, actomyosin clearly still oscillates (Figure 2G), indicating that the coupling between Anillin and actomyosin is crucial for the system to function, as predicted by the conceptual model (Figure 4). In other words, the Anillin N-terminus ensures that Anillin is at the right place (at the CR front) to be able to sequester the appropriate membrane microdomains needed to reduce tension and dissassemble the ring. In the case of Anillin-ΔN, the ectopic, uncoupled formation of anillo-septin occurs at membrane that already trails the CR front. It therefore cannot regulate the tension and closure of the CR front, which is essentially depleted of Anillin. Therefore actomyosin-driven compression of membrane microdomains causes tension to mount to above the usual threshold, resulting in furrow oscillations (Figure 6B).

### The Anillin N-Terminus: A Scaffold for the Actomyosin Network as Well as a Regulator of Anillo-Septin Assembly

According to the model for CR closure (Figure 4), its amino terminus brings Anillin to the mature CR front and is required to position Anillin appropriately for the tension-regulated assembly of the Anillo-septin sub-network that is deposited just behind the CR. However, the N-terminus of Anillin likely also directly scaffolds the actomyosin sub-network, through its domains for binding F-actin, myosin, and Citron kinase (Figure 1), thereby regulating actomyosin contractility. In *Drosophila* S2 cells, expression of the Anillin N-terminus (Anillin-ΔC) suppressed CR oscillations that can occur upon Anillin depletion, but CR closure was still greatly slowed and the furrow membrane exhibited excessive membrane blebbing (Kechad et al., 2012). Similarly, depletion of the septin, Peanut, did not cause furrow oscillations on its own, but it did slow the rate (and limited the full extent) of furrowing (Hickson and O’Farrell, 2008b; Kechad et al., 2012). These results argue that furrow oscillations (and by extension CR tension imbalance) induced by loss of Anillin cannot solely be due to a failure to coordinate anillo-septin assembly with actomyosin, and suggest that Anillin N-terminus-dependent scaffolding of actomyosin also plays a role in furrow stability and therefore tension regulation. Indeed, our prior work suggests that Citron kinase/Sticky contributes to this actomyosin-scaffolding role of the Anillin N-terminus, since Sticky is required for robust recruitment of Anillin-ΔC, and co-depletion of Sticky and the septin Peanut induced unstable furrows reminiscent of Anillin depletion, a phenotype not observed upon single depletions (El Amine et al., 2013). Therefore, Anillin plays a dual role in regulating furrow stability, by regulating both components of the tension balance: actomyosin contraction and intra-membrane resistance to compression.

Actomyosin crosslinking by the Anillin N-terminus may dampen actomyosin contractility at the CR front, so that loss of septin-mediated membrane removal (in the context of Anillin-ΔC) is better tolerated than full depletion of Anillin (less of a tension imbalance). Complete loss of Anillin should accordingly have both enhanced contractility and a reduced means to remove membrane. Failure to remove membrane also enhances contractility by reducing the ability of actomyosin to disassemble. This is consistent with the Anillin-depleted CRs being under the greatest tension and therefore the most prone to oscillation. However, we note that other interactions that Anillin participates in are likely also important for furrow stability, for example those with Tum/RacGAP50c, microtubules and importins (Sisson et al., 2000; D’Avino et al., 2008; Gregory et al., 2008; Hickson and O’Farrell, 2008a; Silverman-Gavrila et al., 2008; Beaudet et al., 2017, 2020).

## Model Summary

### Stage 1: Actomyosin-Dependent CR Assembly

• Rho activation at the cell equator drives inward cortical flow of actomyosin-bound microdomains in the “perpendicular-to-the-ring” axis.
• Anillin is bound to actomyosin through its N-terminus (but not to the membrane).
• Cortical flow and contraction of actomyosin in the circumferential, “around-the-ring” axis compresses actomyosin-bound microdomains.
• Resistance to compression of the membrane microdomains generates tension.
• The CR “matures” at a certain density of microdomains, and threshold in tension, and shifts from the assembly stage to the closure/disassembly stage.

### Stage 2: Actomyosin- and Anillo-Septin-Dependent CR Closure and Disassembly

• Localized compression of actomyosin triggers Anillin disengagement from actomyosin.
• Freed Anillin binds directly to Rho-GTP at the plasma membrane and recruits septins via its C-terminus. This occurs on a membrane microdomain transiently liberated by actomyosin.
• Newly assembled anillo-septin sequesters its membrane microdomain and incorporates into an anillo-septin filament growing in the “perpendicular-to-the-ring” axis.
• Anillo-septin-bound microdomains exit the CR as actomyosin-bound membrane microdomains squeeze past.
• Relative outflow of anillo-septin-bound microdomains releases tension, promotes actomyosin disassembly and allows CR closure.
• Ratchet-like cycles of actomyosin contraction coupled to anillo-septin-bound membrane outflow ensure CR closure and disassembly at the tension threshold.

## Discussion

### Tension Generation Within the CR

Contractile rings must generate tension from forces acting in opposition to each other. The underlying mechanisms of tension generation at CRs have long been elusive, but most consideration has been paid to how the cytoskeletal elements of the CR interact with each other. The other key component of CRs, the plasma membrane itself, seems to have been overlooked as a significant potential contributor to tension generation (besides drag forces) because of the pervasive assumption that membranes are always fluid and always readily deformable by the underlying cytoskeleton. While this assumption may be generally true in many contexts, we argue that it is categorically false in the case of the CR. Indeed, the central tenet of the current model is that the membrane of the CR is anything but compliant: it resists compression by actomyosin contractility, thereby generating tension, and must be actively managed to permit CR closure. Tension generation during CR assembly results largely from the opposing forces of actomyosin contractility and intra-membrane resistance to compression, analogous to the tension holding a wine barrel together.

If CR tension is a balance between cytoskeletal contraction and intra-membrane resistance to compression, then Anillin appears to operate on both sides of that balance. It dampens contractility (both through scaffolding and promotion of depolymerization by membrane sequestration) and reduces resistance to compression of the membrane (through membrane outflow). Thus Anillin reduces the tension required for CR closure, acting like molecular grease. Without Anillin, higher tension results (and is needed) because of both high contractility and high resistance to compression.

This view might explain why different levels of Anillin either speed up or slow down CR closure in *C. elegans* zygotes, with intermediate levels promoting the fastest rate (Descovich et al., 2018). Different levels of Anillin might influence the two sides of the tension-balancing system slightly differently. *C. elegans* zygote CRs still complete ingression after anillo-septin depletion, but the rate of closure is slowed and higher levels of myosin are required (Maddox et al., 2007). Furthermore, this elevated myosin appears to spread more uniformly around the ring (Maddox et al., 2007). Thus, these anillo-septin-depleted CRs require more actomyosin contractility to close, presumably under higher tension. Anillo-septin depletion in the same cells also leads to enhanced recruitment of an F-actin probe to the CR (Jordan et al., 2016), which may reflect impaired actomyosin disassembly. Intriguingly, Anillo-septin depletion was also shown to rescue cytokinesis failure in temperature-sensitive formin mutants (Jordan et al., 2016). In light of our model, it is tempting to speculate that formin-deficient CRs might be defective at generating and maintaining tension when wild-type anillo-septin is present because the latter removes the CR membrane microdomains too effectively, causing tension to drop below a minimum threshold for CR closure. Conversely, anillo-septin depletion might allow formin-deficient CRs to close successfully by making them reliant on a less-effective mechanism of membrane removal, such that the defective actomyosin sub-network can now sustain enough tension to maintain CR integrity. Collectively, these data appear to support, and do not refute, the view that a balance between actomyosin-driven membrane compression and anillo-septin-dependent membrane removal are central to tension control within the CR, with the former sub-network building tension, the latter dissipating it.

The proposed intra-membrane resistance to compression can explain other diverse phenomena, such as why CRs isolated from fission yeast “ghosts” (permeabilized protoplasts lacking a cell wall) display sections of actomyosin that are not anchored to the membrane and that shorten at a rate 30 times faster than that of adjacent, membrane-anchored sections (Jochova et al., 1991; Mishra et al., 2013; Stachowiak et al., 2014; Wang and O’Shaughnessy, 2019). It also can explain why CR closure is much slower than one would predict from the velocities of load-free myosin *in vitro* (Stark et al., 2010; Mishra et al., 2013; Alonso-Matilla et al., 2019). In both cases, the intra-membrane resistance to compression of the CR-anchored membrane microdomains may impede actomyosin contractility.

### The CR Is “*Consumed by Its Own Contractility*”

Once formed, how does a mature CR close? Again the poorly understood connections to the plasma membrane are seldom considered. However, Schroeder recognized their central importance and, based on the rapidity of the disruptive effects of the actin inhibitor cytochalasin B, surmised that “*individual filament-membrane attachments are firm but of brief duration*” (Schroeder, 1990). Schroeder also noted from his seminal electron microscopy studies that “*the furrow membrane neither folds nor buckles, and any microvilli in the furrow do not become especially concentrated, suggesting that they migrate out. Evidently, therefore, membrane “flows” or shears away from the bottom of the furrow out to the walls or shoulders, leaving the contractile ring behind. Somehow the contractile ring maintains its mechanical purchase on the membrane despite a “fluid” attachment*” (Schroeder, 1990). Our model is entirely consistent with Schroeder’s visionary inferences: the Rho-dependent anillo-septin sub-network provides the means for the membrane attachment sites to flow out from the CR, thereby relaxing the tension built-up by actomyosin contractility. Our live imaging observations indicate that the actomyosin network can effectively “step over” the membrane-bound anillo-septin structures formed by the anillin C-terminus (Anillin-ΔN), since it neither drags them along as it oscillates, nor is it slowed down as it passes (Kechad et al., 2012; Figure 2G). This is consistent with dynamic on/off binding of the plasma membrane by actomyosin, which is also supported by the rapidity of the effects of actin drugs (Schroeder, 1972; Hickson and O’Farrell, 2008b), as well as numerous FRAP studies indicating that CR components turn over rapidly (seconds) (Guha et al., 2005; Murthy and Wadsworth, 2005; Uehara et al., 2010; Kondo et al., 2011; Beaudet et al., 2020). Indeed, at the mature CR we envision membrane microdomains shuttling from the actomyosin sub-network to the anillo-septin network, where they are sequestered and deposited behind the CR as it continues its onward journey, while closing at the same time. This proposed contraction-coupled outflow of anillo-septin-bound membrane microdomains finally provides a plausible mechanistic explanation for how the CR sheds its components (by bifurcated disassembly) and is “*consumed by its own contractility*” (Schroeder, 1972).

### Composition of Membrane Microdomains and Protein Anchors

For the CR membrane attachment sites to be able to resist compression induced by actomyosin contractility and/or crosslinking they need to be of sufficient size and rigidity. We envision them as membrane microdomains, such as cholesterol- and sphingolipid-rich lipid rafts (Lingwood and Simons, 2010; Neto et al., 2011). Several observations suggest the importance of lipid rafts to cytokinesis: cholesterol and other raft components accumulate during cytokinesis (Ng et al., 2005; Atilla-Gokcumen et al., 2014; Makino et al., 2015), and cytokinesis is impaired upon depletion of these components (Feng et al., 2002; Fernandez et al., 2004; Szafer-Glusman et al., 2008; Atilla-Gokcumen et al., 2011, 2014; Abe et al., 2012).

The phosphoinositide phosphatidyl-inositol (4,5) bisphosphate (PIP2) is a strong candidate receptor at the plasma membrane that is known to bind many components of the cortical cytoskeleton (Logan and Mandato, 2006). Although PIP2 localization to lipid rafts is still debated, PIP2 can form clusters with cations, basic peptides and other lipids (Wang et al., 2014; Brown, 2015), and has been observed in 64 nm wide membrane microdomains as measured by stochastic optical reconstruction microscopy (STORM) (Wang and Richards, 2012). PIP2 accumulates in the cleavage furrows of human cells (Field S.J. et al., 2005), in a manner that depends on cholesterol and sphingomyelin (Abe et al., 2012). Perturbing PIP2 through overexpression of PIP2-binding domains or PIP2 phosphatase decreased RhoA levels at the furrow and caused separation of F-actin from the plasma membrane, consistent with a role for PIP2 in anchoring the CR (Field S.J. et al., 2005; Abe et al., 2012).

Anillin and septins are known to bind to PIP2 (Tanaka-Takiguchi et al., 2009; Bertin et al., 2010; Liu et al., 2012). Conversely, the mechanisms by which actomyosin are anchored to the CR membrane are poorly understood, although myosin II can localize to the plasma membrane independently of F-actin and Anillin in fly and human cells (Dean et al., 2005; Kamijo et al., 2006; Hickson and O’Farrell, 2008b). PIP2 is an attractive candidate membrane component for binding both the actomyosin and anillo-septin sub-networks. However, it is also conceivable that different lipids/proteins within the microdomains, which are likely to have a heterogeneous composition (Neto et al., 2011; Echard, 2012; Atilla-Gokcumen et al., 2014), could bind different components of each sub-network (actomyosin versus anillo-septin) while still maintaining mutually exclusive binding. Despite the undoubted complexity of the lipid composition of the CR membrane (Neto et al., 2011; Echard, 2012; Atilla-Gokcumen et al., 2014), our model makes the simple prediction that, at the mature CR, discrete membrane microdomains switch from the actomyosin sub-network to the anillo-septin sub-network, allowing furrow advancement (in the “perpendicular-to-the-ring” axis) and CR closure (in the “around-the-ring” axis).

Although we refer to actomyosin as one entity for simplicity and clarity, this sub-network must itself be anchored to the membrane through at least two mechanisms: one for F-actin and one for myosin, and likely multiple others. Further elaboration of the concept will therefore require the consideration of additional microdomain attachments: for F-actin, myosin and anillo-septin. Rho-kinase and Citron kinase/Sticky are also present and presumably bound to the membrane, although the latter is not required for CR closure in *Drosophila* S2 cells, and nor is the former so long as myosin is active (Echard et al., 2004; Dean and Spudich, 2006; Hickson et al., 2006). The anillo-septin-independent mechanisms of membrane anchoring of actomyosin likely involve direct lipid binding in the case of myosin (Li et al., 1994; Murakami et al., 1994, 1995; Liu et al., 2016) and the formin, Diaphanous, in the case of F-actin (Ramalingam et al., 2010; Bucki et al., 2019). Diaphanous-related formins can bind and cluster PIP2 within cholesterol microdomains (Bucki et al., 2019). Other proteins may also participate as membrane anchors linking actomyosin to the plasma membrane independently of anillo-septin. Candidates include ezrin-radixin-moesin (ERM) family proteins that bind both F-actin and PIP2 and maintain cortical stability during mitosis and cytokinesis (Carreno et al., 2008; Roubinet et al., 2011; Kunda et al., 2012; Hiruma et al., 2017). Interestingly, Anillin depletion in human cells has been shown to enhance ERM localization to the furrow, although ERMs did not track well with the resulting oscillating furrows (Hiruma et al., 2017). Supervillin, which can bind F-actin, myosin and the plasma membrane, is another candidate auxilliary anchor protein that acts during cytokinesis (Pestonjamasp et al., 1995, 1997; Fang et al., 2010; Smith et al., 2010, 2013; Hiruma et al., 2017).

### Anillo-Septin Regulation and Function

The proposed model has a number of implications regarding the regulation and function of Anillo-septin. Firstly, although Anillin is well known to bundle and cross-link actomyosin, this model highlights the fact that actomyosin crosslinking is not the only important role for Anillin at the CR. The robust and polymorphic interactions between Anillin and actomyosin (Field and Alberts, 1995; Straight et al., 2005; Jananji et al., 2017; Matsuda et al., 2020) also serve to ensure that Anillin molecules are always appropriately positioned at the right place and time to effectively sequester membrane microdomains from actomyosin at the CR to release tension. Anillin-actomyosin interactions are also important for regulating the later transition to the midbody ring, after CR closure, which is coupled to actin disassembly and requires the Anillin N-terminus (Kechad et al., 2012; El Amine et al., 2013).

Although septin−myosin interactions have been reported in human cells (Joo et al., 2007) and septin−actin interactions are clearly observed in *Drosophila* embryos and *in vitro* (Mavrakis et al., 2014), our observations and model suggest that, at least at the CR of S2 cells, septins are linked to F-actin and myosin only via Anillin, if at all. Septins are clearly not recruited to actomyosin rings depleted of Anillin in *Drosophila* S2 cells or *C. elegans* zygotes (Maddox et al., 2005, 2007; Hickson and O’Farrell, 2008b). Indeed, as elaborated above, we propose that Anillin is bound either to actomyosin or to septin, but not to both at the same time (at least at the mature CR). This may seem counter to observations in human cells, where Anillin has been shown to recruit septins to actin bundles (Kinoshita et al., 2002), but those cells were in interphase, the actin bundles cytoplasmic, and Rho was likely not involved. Clearly, further work is needed to understand the full complement of interactions between septins, actomyosin, and Anillin, and how Rho and membranes modulate them throughout cytokinetic progression.

Septins have been shown to bind preferentially to membranes of a positive, micron-scale curvature (Bridges et al., 2016; Beber et al., 2019; Cannon et al., 2019; McMurray, 2019). Although the CR undoubtedly harbor regions of changing membrane curvature, the Rho/Anillin-dependent recruitment of septins to the plasma membrane that we have observed occurs just as effectively in cells treated with LatA (where no such curvature exists) as in cells with a constricting actomyosin ring. Indeed in LatA, Rho1, Anillin and septins form tubular structures that envelop themselves in membrane: i.e., that membrane becomes curved (Hickson and O’Farrell, 2008b). Perhaps Rho/Anillin binding induces septin rods and/or filaments to adopt a particular curvature that enhances membrane binding, while promoting higher-order assemblies capable of inducing membrane deformation?

We view the primary role of anillo-septin assembly as a robust mechanism to sequester membrane from actomyosin, opposing its contractility so that tension can be maintained around an optimal threshold. In cells treated with LatA (i.e., without tension), anillo-septin forms tubular structures (Figures 2A,B; Hickson and O’Farrell, 2008b). It seems most logical to consider that these structures represent unrestricted anillo-septin assembly forming supramolecular coils via septin polymerization at their base, zippering up membrane microdomains as they assemble. In the absence of F-actin, these putative coils represent the relaxed state of anillo-septin, like springs under zero tension. However, in the presence of F-actin, we envision that nascent anillo-septin filaments that trail the advancing CR to which they are connected are being stretched out under tension by the advancing CR, as depicted in Figure 5C. Accordingly, we predict that the anillo-septin filaments that project radially out from the CR accumulate stored energy and a spring-like quality, ready to recoil back up the flanks of the furrow upon disconnection from the CR front. Such a recoil mechanism could be dependent on anillo-septin filament length (and therefore age) since tension along the anillo-septin filament could increase with length (and age). Such a putative recoil mechanism would facilitate anillo-septin-dependent membrane microdomain removal from the closing CR, thereby promoting local reduction of tension in the “around-the-ring” axis. Tensile springs of anillo-septin filaments could also rigidify the furrow membrane and stabilize its ingression. This hypothetical mechanism can explain why the furrows of cells depleted of anillo-septin exhibit excessive membrane blebbing (Somma et al., 2002; Kechad et al., 2012; El Amine et al., 2013) and are prone to oscillation. It can also begin to explain our observations that anillo-septin drives the extrusion and shedding of plasma membrane tubules from the nascent midbody ring as the CR F-actin disassembles (El Amine et al., 2013).

Although our model predicts that actomyosin and anillo-septin are not directly connected with one another, an important concept arises in which the two sub-networks can influence one another indirectly through their underlying membrane microdomains. The radially organized, anillo-septin-bound membrane microdomains emanating from the CR into the flanks of the furrow should be relatively static (at least until the predicted outward recoil) and could therefore form the walls of channels within the lipid bilayer, through which actomyosin-anchored membrane microdomains can flow into the CR as they advance it (Figure 5). This concept seems relevant to the enigma of the hourglass-to-double ring transition of septin filaments that has been described for budding yeast CRs (Vrabioiu and Mitchison, 2006), and that is regulated by the anillin-like Bud4 (Ong et al., 2014; Chen X. et al., 2020). Intra-membrane flow of microdomains anchored to actomyosin CR components could help orient the differentially membrane-anchored septin filaments of the hourglass, while a reduction/cessation of that flow could allow a 90-degree rotation of the septin filaments to a “preferred” circumferential arrangement.

### The Rho Network Micro-Manages Its Own Membrane Microdomain Attachment Sites

What makes the contraction-coupled membrane sorting model particularly compelling is that both actomyosin and anillo-septin sub-networks are directly controlled by the Rho GTPase. This means that the system can be wired to locally and intrinsically balance actomyosin contractility (which drives CR assembly) with relative anillo-septin membrane outflow (which drives CR disassembly and closure). However, this raises additional, intriguing questions regarding how the Rho GTPase might control the system.

A recent series of creative experiments using engineered chimeric proteins expressed at cell junctions and cleavage furrows of MCF-7 cells, has led Yap and colleagues to propose that the human Anillin AH domain is required to promote actomyosin activity by cyclically “resetting” Rho-GTP at the membrane, dynamically binding and releasing it, thereby prolonging its cortical residency in a manner that promotes activation of effectors for actomyosin assembly (Budnar et al., 2019; Munro, 2019; Morris et al., 2020). However, some aspects of that model appear incongruous with ours. Firstly, Anillin-depleted furrows do not appear to be defective in activating actomyosin effectors in *Drosophila* S2 cells (Hickson and O’Farrell, 2008b; Kechad et al., 2012), or HeLa cells (Straight et al., 2005; Zhao and Fang, 2005; Piekny and Glotzer, 2008). Secondly, actomyosin and anillo-septin (Anillin-ΔN) become so spatially segregated from one another (Piekny and Glotzer, 2008; Kechad et al., 2012) that it is difficult to imagine how they could share a pool of Rho as proposed by Budnar et al. In S2 cells, Rho1 is enriched in Anillin-ΔN structures, LatA-induced structures and in the anillo-septin-dependent membranes extruded from the nascent MR (Hickson and O’Farrell, 2008b; Kechad et al., 2012; El Amine et al., 2013). Although we have not directly monitored the Rho1 effectors Dia and Rok, one assumes they would track with actomyosin, which is excluded from the anillo-septin-specific structures.

Rather than Anillin sharing Rho1-GTP with effectors for actomyosin assembly, our model is more consistent with sequential transfer of Rho1 from actomyosin effectors to Anillin. This latter idea fits well with the current model’s proposed sequential transfer of membrane microdomains from actomyosin to anillo-septin, but it rather implies that anillo-septin sequesters Rho1 and membrane away from actomyosin (but only after actomyosin has been activated and served its purpose).

Anillin reportedly binds Rho-GTP with much less affinity than other effectors (Blumenstein and Ahmadian, 2004; Sun et al., 2015), yet it is clearly very competent to access Rho-GTP at the equatorial membrane, even when many of its other modes of recruitment are absent, as exemplified by Anillin-AHPH (Anillin C-terminus), which can no longer bind F-actin, myosin or Citron kinase/Sticky. The recruitment of the Anillin-AHPH is Rho-dependent and Anillin itself is required for the major fraction of detectable RhoA at the CR of human cells (Piekny et al., 2005; Piekny and Glotzer, 2008; Budnar et al., 2019). Thus perhaps Anillin sustains a pool of Rho-GTP and PIP2, as elegantly shown by Budnar et al. (2019), not so much to promote actomyosin activation, but rather to prolong the lifetime of the Anillo-septin sub-network itself, as it trails behind the advancing actomyosin. According to this view, Anillo-septin-bound Rho might reflect a temporal record of Rho activation, with “new” Rho-GTP closest to the CR and “old” Rho-GTP emanating further into the flanks of the furrow (rather like the vapor trails marking the passage of an aircraft). This can explain why furrow-associated Rho levels are much lower in cells depleted of Anillin (Piekny and Glotzer, 2008). Actomyosin contractility might persist in Anillin-depleted furrows, because actomyosin is ordinarily always activated first, ahead of Anillin. Anillin may then be positioned to accept the Rho-GTP, presumably as Anillin disengages from actomyosin and assembles onto the switching membrane microdomains. This view implies that Rho may be sequentially transferred through its different effectors, starting with the actomyosin activators and ending with anillo-septin. The RhoGEF ECT2/Pebble, which activates the entire Rho-dependent network, also needs to access the membrane to function (Kotynkova et al., 2016). A recent structural study indicates that allosteric binding of Rho-GTP to the ECT2 PH domain is needed to relieve autoinhibition on the adjacent DH domain and fully activate its GEF activity (Chen M. et al., 2020). Conceivably, as the “older” Rho-Anillo-septin network begins to break down (and/or is stretched out to a certain point prior to recoil), it could release some Rho-GTP to re-activate ECT2/Pebble and/or actomyosin. Thus Anillin-dependent prolongation of Rho-GTP lifetime, as shown by Budnar et al. (2019), could potentially act as a controlled-release mechanism to sustain an expanded Rho-zone behind the CR. This could help maintain the anillo-septin sub-network and boost cortical/membrane flow back in toward the CR to maintain the appropriate tension there. Anillin-dependent positive feedback on Rho activity has been proposed before (Piekny and Glotzer, 2008; Frenette et al., 2012; Budnar et al., 2019), but this putative time-resolved mechanism fits particularly well with the analogy that the CR comprises self-sustaining conveyor belts, or “caterpillar^TM^ tracks,” of membrane microdomains that are continually flowing in (with actomyosin) and out (with anillo-septin) in the “perpendicular-to-the-ring” axis, with net loss of material from the CR front as it shrinks in the “around-the-ring” axis.

### Membrane Growth During CR Closure

For cleavage furrows to ingress, the plasma membrane must “grow” inward toward the cell interior, yet where the membrane comes from and how the membrane growth occurs has been far from clear. There is an extensive body of literature suggesting that the addition of new membrane (i.e., from internal stores) is required for furrow ingression (Bluemink and de Laat, 1973; de Laat and Bluemink, 1974; Shuster and Burgess, 2002) and/or completion of cytokinesis (reviewed in Albertson et al., 2005; Prekeris and Gould, 2008; Neto et al., 2011; D’Avino et al., 2015; Fremont and Echard, 2018; Gerien and Wu, 2018). Membrane delivery by exocytosis can certainly occur in the flanks of the furrow to increase surface area, for example through polarized secretion via the exocyst complex (Giansanti et al., 2015). However, addition of membrane by exocytosis to the CR front seems incompatible with the density of components and tension there, and there is no good evidence for it (e.g., Schroeder’s electron micrographs). Rather, we propose that the membrane ingresses in an orderly fashion by being physically reeled in by the contracting CR (in the “around-the-ring” axis), while being compressed by cortical flow (in the “perpendicular-to-the-ring” axis).

### Implications for Controlling the Rate of CR Closure

A recent study of *C. elegans* zygote cytokinesis by Khaliullin et al. (2018) showed that CRs accelerate their per-unit-length constriction rate as they close, and in a manner that can be explained by an exponential increase in Rho network components (such as myosin and Anillin). Based on these experimental data and mathematical modeling a “constriction-coupled disassembly with compression feedback” model was proposed, in which actomyosin cortical flow promotes more cortical flow through positive feedback (Khaliullin et al., 2018). While bearing similarity to the current proposal in terms of cortex compression, that study did not consider the overlying plasma membrane, which is central to the proposed contraction-coupled membrane microdomain sorting model. This posits that actomyosin-driven membrane microdomain flow into the CR is balanced by relative outflow of anillo-septin-bound membrane microdomains, through tension-regulated membrane microdomain switching events (from actomyosin to anillo-septin). The membrane inflow promotes furrow advancement in the “perpendicular-to-the-ring” axis while relative outflow of promotes CR closure in the “around-the-ring” axis. Increased rates of per-unit-length constriction of the “around-the-ring” axis might therefore reflect more frequent membrane microdomain switching events occurring at higher tension (or at higher tension production potential). Such conditions might also cause microdomain switching events to occur further back into the “perpendicular-to-the-ring” axis, promoting faster removal of membrane in the “around-the-ring” axis and faster advancement in the perpendicular axis. The deposited anillo-septin may in turn promote additional cortical flow through releasing Rho-GTP in an expanded Rho-zone, while its underlying membrane domains provide conduits to channel actomyosin-bound membrane domains forward. We also note that centralspindlin, required to activate the RhoGEF Pebble/ECT2 at the membrane, becomes increasingly concentrated there as the furrow ingresses (see Figure 3A). This may also expand the Rho zone to accelerate the rate of CR closure.

It has also been shown in early *C. elegans* embryos that larger cells close their CRs at a faster rate than their smaller progeny, such that the total time of furrowing stays constant (Carvalho et al., 2009). Larger cells presumably have a greater pool of centralspindlin/ECT2 such that, as furrowing progresses, their spindles and furrows will accumulate a more concentrated bolus of the Rho activation machinery than smaller cells do. Larger cells may also build more tension in their CRs sooner than small cells because of a more expansive cortical network with which to fuel the cortical flow required for generating and sustaining tension.

### Relevance to the Symmetry of CR Closure and Unilateral Furrows

A widespread property of CRs is their tendency to close asymmetrically, such that the center of the CR (viewed down the spindle/“perpendicular-to-the-ring” axis) is displaced during closure (Maddox et al., 2007). In the most extreme cases, unilateral furrows can ingress from one side of the cell before the CR is fully assembled (e.g., Rappaport and Conrad, 1963; Savoian et al., 1999). In *C. elegans* embryos, the asymmetry of closure was shown to require Anillin and septins (Maddox et al., 2007), since Anillin (ANI-1) or septin (UNC-59 and UNC-61) depletion led to symmetric closure. Furthermore, these anillo-septin-deficient CRs were shown to be sensitive to reductions in myosin levels/activity that are usually tolerated (Maddox et al., 2007). Myosin levels increase in anillo-septin-deficient rings (see Figure 3B in Maddox et al., 2007). Mathematical modeling has led to the suggestion that feedback among membrane curvature, cytoskeletal alignment, and contractility is responsible for asymmetric CR closure (Dorn et al., 2016). However, an alternative model has also been proposed in which radially asymmetric cortical flows account for the asymmetric closure (Menon et al., 2017). This model is supported by the observations that cortical flows are sufficient to align actin filaments in the circumferential, “around-the-ring” axis and are attenuated in anillin/ANI-1-depleted embryos (Reymann et al., 2016). Our observations and model are also consistent with this view and further predict that radially asymmetric outflow (i.e., non-uniform around the CR) of anillo-septin-membrane microdomains is a driving factor also, that should go hand-in-hand with asymmetric cortical inflow (actomyosin-dependent), since both are co-dependent on active Rho (Michaux et al., 2018). The tight coupling between actomyosin contraction and anillo-septin outflow could amplify any local asymmetries in Rho activation or in resistance within the membrane, or both since they are likely inter-dependent. In the case of a mature CR, if tension were evenly maintained throughout the ring (which is not necessarily the case), then membrane microdomains would only need to flow out of the region of least resistance to promote closure. And once outflow starts in a given region, the same region might continue to offer the least resistance leading to asymmetric closure of the CR.

The proposed membrane microdomain compression and sorting mechanism can accommodate conditions where there is no complete ring, such as rings cut with lasers, which recover and close (Silva et al., 2016), and unilateral furrows. Indeed, because the proposed tension regulation occurs on membrane microdomains that are compressed between those anchored to contracting actomyosin, it should be able to operate at any discrete region of cortex where Rho is locally activated. Indeed optogenetic activation of RhoA anywhere at the plasma membrane of HeLa cells is sufficient to initiate furrowing (Wagner and Glotzer, 2016), including unilateral furrowing (Kotynkova et al., 2016). The model supports the view that the CR should not really be considered as a single entity, but rather as a collection of (semi)-independent contractile modules that congregate into a ring and contract (semi)-autonomously yet in unison (Silva et al., 2016).

### Relevance to Other Species and Processes

While mechanistic details likely exist between systems and species, we propose that regulated membrane microdomain micromanagement will be a conserved feature of CR assembly, tension regulation and closure. Indeed the concept of tension generation through cytoskeleton-regulated membrane microdomain compression may also be relevant to diverse, yet mechanistically related processes such as apical constriction and would healing. Apical constriction of epithelial cells proceeds via a ratchet-like mechanism driven by pulsed actomyosin contractions (Martin et al., 2009; Mason et al., 2013; Acharya et al., 2017). Epithelial wounds heal via contraction of a supracellular actomyosin cable that forms at the wound edges (Rothenberg and Fernandez-Gonzalez, 2019), and involves collaboration between actomyosin and septins (Shindo et al., 2018). Considering these processes from the perspective of the membrane microdomains to which the cytoskeleton is attached, as we have done here, will likely yield important mechanistic insight. Single-cell wound healing, as observed in *Xenopus* oocytes and syncytial *Drosophila* embryos (Bement et al., 1999, 2006; Abreu-Blanco et al., 2011), is another process particularly analogous to cytokinesis. After an initial rapid resealing of the ruptured plasma membrane by exocytosis, an actomyosin-based CR forms around the wound site, in a structure resembling the cartoon of Figure 5B (McNeil et al., 2000; Boucher and Mandato, 2015; Davenport et al., 2016). This CR then closes over several minutes, driven by cortical flow powered by an analogous Rho-dependent network. These CRs are under substantial tension as evidenced by the observation that flow-transported microtubules buckle and break as they slam into the CRs (Mandato and Bement, 2003). Yet, it remains unclear why the rings close so slowly, especially if the membrane is already sealed. In light of our model, it seems logical to suggest that the tension reflects resistance to compression of the underlying membrane microdomains that become jammed into a ring within the bilayer at the wound site. Accordingly, closure of these CRs could depend on an analogous sorting of membrane microdomains, allowing them to slide/jostle past one another and invade the virgin membrane (devoid of appropriate microdomains) that temporarily plugged the wound. Thus, we suggest that cytoskeletal micromanagement of membrane microdomain attachment sites may be key for diverse contractile arrays beyond cytokinesis (Bement, 2002, Bement et al., 2006).

## Conclusion

In summary, we propose that the widely accepted concept of the Rho-dependent actomyosin CR be extended to include the Rho-dependent actomyosin-anilloseptin, membrane-anchored ring (the AMAS ring), controlled by Rho and organized by Anillin. The AMAS ring buffers its own tension, building it through actomyosin gathering and compressing the CR’s membrane microdomain attachment sites and releasing it through anillo-septin removing the CR’s membrane microdomains. The AMAS ring has two stages: assembly and disassembly/closure. Assembly occurs by compressing membrane microdomains within the plane of the bilayer in the “perpendicular-to-the ring” axis, generating tension. Closure then occurs by bifurcated disassembly, whereby ratchet-like cycles of actomyosin contraction in the “around-the-ring” axis squeeze anillo-septin-bound microdomains out of the ring in the “perpendicular-to-the ring” axis, thereby regulating tension, reducing the circumference of the ring and promoting net depolymerization of actomyosin, all at the same time. Considering the dynamic properties of this more inclusive AMAS ring, which places crucial emphasis on the membrane microdomain attachment sites, provides a novel and detailed conceptual framework for understanding the inner workings of CRs. This concept needs to be rigorously tested using a variety of systems and techniques from cell biology to physics and mathematical modeling.

**FIGURE 7:**
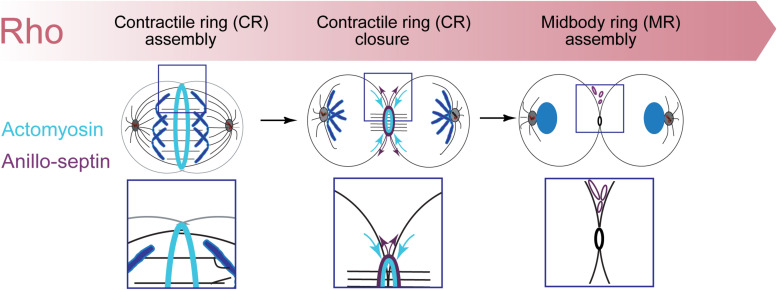
The Rho-dependent network drives cytokinetic progression from CR assembly, through closure/disassembly and to midbody ring assembly.

Finally, in considering the different Rho-dependent sub-networks (Figure 1), our observations also provide insight into the bigger picture view of how cytokinetic ring structures mature during successive stages of cytokinetic progression. The actomyosin sub-network drives CR assembly and closure, while the anillo-septin sub-network promotes CR closure and disassembly. Finally, an Anillin-Citron kinase sub-network promotes MR formation (Figure 7). Thus, maturation of the cell cortex during cytokinesis reflects a shifting importance of different sub-networks controlled by the master activator, Rho, and coordinated by the master organizer, Anillin. Mapping how these different sub-networks are integrated with one another throughout cytokinetic progression remains an important challenge.

## Data Availability Statement

All datasets generated for this study are included in the article.

## Author Contributions

GH wrote the manuscript, with significant input from SC and AK, who both contributed equally to the conceptualization and refinement of the proposed hypothesis and how it fits with published literature. GH and SC wrote the abstract. AK assembled the Figures 1, 7. SC assembled the Figures 2–6. All authors read and edited the submitted version of the manuscript.

## Conflict of Interest

The authors declare that the research was conducted in the absence of any commercial or financial relationships that could be construed as a potential conflict of interest.
